# Cellular reprogramming for clinical cartilage repair

**DOI:** 10.1007/s10565-017-9382-0

**Published:** 2017-01-31

**Authors:** Britta J.H. Driessen, Colin Logie, Lucienne A. Vonk

**Affiliations:** 1 Department of Orthopaedics, University Medical Center Utrecht, Utrecht, The Netherlands; 2 Department of Molecular Biology, Radboud Institute for Molecular Life Sciences, Nijmegen, The Netherlands

**Keywords:** Articular cartilage, Clinical application, Direct lineage reprogramming, Induced pluripotent stem cells, Regenerative medicine

## Abstract

The repair of articular cartilage needs a sufficient number of chondrocytes to replace the defect tissue, and therefore, expansion of cells is generally required. Chondrocytes derived by cellular reprogramming may provide a solution to the limitations of current (stem) cell-based therapies. In this article, two distinct approaches—induced pluripotent stem cell (iPSC)-mediated reprogramming and direct lineage conversion—are analysed and compared according to criteria that encompass the qualification of the method and the derived chondrocytes for the purpose of clinical application. Progress in iPSC generation has provided insights into the replacement of reprogramming factors by small molecules and chemical compounds. As follows, multistage chondrogenic differentiation methods have shown to improve the chondrocyte yield and quality. Nevertheless, the iPSC ‘detour’ remains a time- and cost-consuming approach. Direct conversion of fibroblasts into chondrocytes provides a slight advantage over these aspects compared to the iPSC detour. However, the requirement of constitutive transgene expression to inhibit hypertrophic differentiation limits this approach of being translated to the clinic. It can be concluded that the quality of the derived chondrocytes highly depends on the characteristics of the reprogramming method and that this is important to keep in mind during the experimental set-up. Further research into both reprogramming approaches for clinical cartilage repair has to include proper control groups and epigenetic profiling to optimize the techniques and eventually derive functionally stable articular chondrocytes.

## Introduction

Articular cartilage is a load-bearing, avascular tissue composed of chondrocytes embedded in an extracellular matrix (ECM) to form the basis of smooth articulation of the joint. Ageing and trauma are the main causes of cartilage degeneration, a common issue in the human population (Heidari 2011). During life, mechanical load on damaged joints can accelerate focal cartilage degeneration, eventually leading to the onset of osteoarthritis. The avascularity of cartilage and mitotically inactive chondrocytes limits the intrinsic healing capacity of the tissue.

Current therapies include microfracture (MFX) for relatively small defects (<2 cm^2^) (Trice et al. 2010) and autologous chondrocyte implantation (ACI) for larger defects. ACI, introduced by Brittberg et al. (1994), is the first clinical approved cell therapy that aims to regenerate functional cartilage (Brittberg et al. 1994). This therapy involves the harvest of chondrocytes from a low weight-bearing area followed by culture expansion to obtain sufficient cells and subsequent reinjection of the cells into the defect. Although the clinical outcome of ACI is satisfactory, the expansion of the chondrocytes affects chondrogenic quality and causes dedifferentiation, i.e. an up-regulation of type I collagen and down-regulation of type II collagen and aggrecan expression which often results in fibrocartilage as repair tissue (Ma et al. 2013a). Also, the intervention is expensive and it requires two surgeries which makes the therapy invasive. ACI is considered to be effective for cartilage defects ranging from 2 to 5 cm^2^; the restoration of defects larger than that remains a challenge (EMA 2014).

Nowadays, multipotent mesenchymal stromal cells (MSCs) are the most well-studied stem cells because of their proliferative features, immunomodulatory characteristics and chondrogenic potential (Pittenger et al. 1999). Unfavourably, limitations of MSCs are the heterogeneity, limited chondrogenic capacity after expansion and age-related function decline (Ho et al. 2008; Lubis and Lubis 2012). Together with the invasive harvesting method and low cell yield, MSC-based cartilage regeneration could be substituted by other stem cell types. This has provided a window of opportunity for induced pluripotent stem cells (iPSCs) to enter the field of cartilage regeneration.

The commonly known iPSCs discovered by Takahashi and Yamanaka (2006) provide a cell source with both the self-renewal capacity and the potential to differentiate into every desired cell type of ecto-, endo-, or mesodermal origin. iPSCs are derived by cellular reprogramming approaches. In general, accessible and abundant differentiated cell types such as fibroblasts are reverted into a pluripotent cell state. The iPSCs are functionally comparable to embryonic stem cells (ESCs) while avoiding the ethical issues related to ESCs (Lo and Parham 2009). However, obtaining iPSCs is not a simple procedure and carries several challenges, such as viral transduction which is based on gene integration, and often results in iPSCs with oncogenic potentials (Tsumaki et al. 2015).

**Fig. 1:**
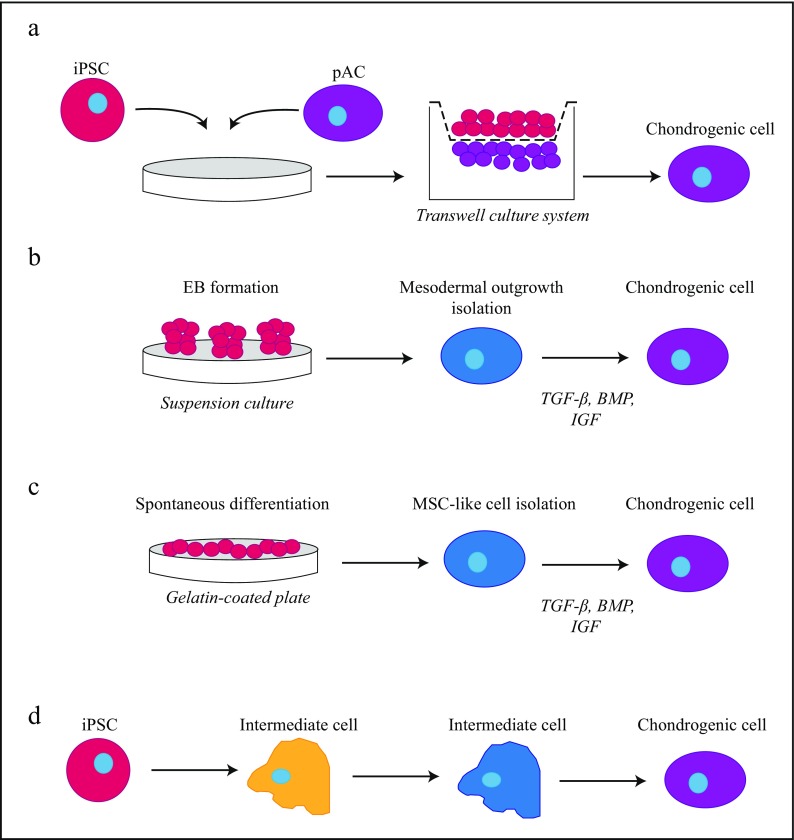
Chondrogenic differentiation methods applied to iPSCs. **a** Chondrogenic differentiation of iPSCs is driven by co-culture with primary articular chondrocytes in a transwell culture system. **b** iPSCs are placed into suspension culture to form embryoid bodies (EBs); mesodermal outgrowth is isolated followed by chondrogenic differentiation with pro-chondrogenic growth factors and cytokines. **c** Spontaneous differentiation of iPSCs is carried out in 2D monolayer on gelatin-coated culture plates, MSC-like cells are isolated, and chondrogenic differentiation is stimulated. **d** Directed chondrogenic differentiation is carried out by segmentation of the differentiation protocol; intermediate cell stages are passed towards a chondrogenic phenotype. Adapted from Oldershaw (2012)

Following iPSC generation, proper differentiation methods are necessary to steer the stem cells into chondrocytes. These methods can be classified in four groups (Fig. 1) and are reviewed by Tsumaki and colleagues (Tsumaki et al. 2015). Although iPSCs seem promising for articular cartilage regeneration, time- and cost-efficiency of the approach are today’s challenges.

Besides iPSC-based reprogramming, direct conversion of fibroblasts into the desired cell type is another approach to obtain a pool of chondrocytes (Ladewig et al. 2013). Direct conversion is based on a similar concept in which cellular reprogramming is central. Chondrocytes, derived by direct conversion, differ from iPSC-derived chondrocytes in the sense that the acquisition of a PSC fate is not involved. In the field of cartilage regeneration, both techniques have proven to be effective in producing chondrocytes (Diekman et al. 2012; Ko et al. 2014; Hiramatsu et al. 2011). However, the efficiency of the methods and the safety and stability of the derived chondrocytes have not been considered and compared in depth. Additionally, it has not yet been clarified which method is most beneficial for the development of chondrocytes with quality standards adequate for clinical use.

This study aims to define the status quo of cellular reprogramming techniques in cartilage regeneration. The development of chondrocytes by direct conversion or through iPSCs is analysed according to several criteria that account for the qualification of the methods and the derived chondrocytes. By this study, we hope to provide insights into the reprogramming technique that is most beneficial for clinical cartilage repair.

## Articular cartilage: development and physiology

**Fig. 2:**
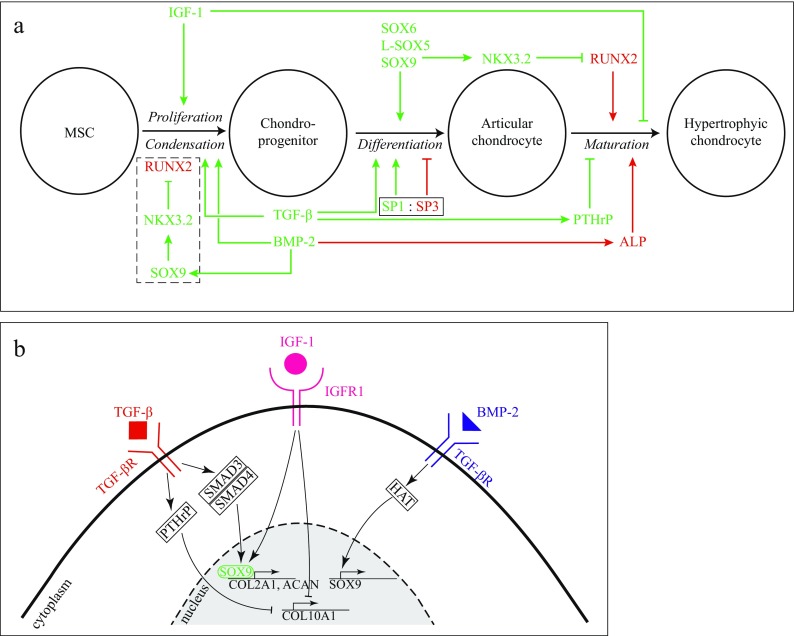
Growth and transcription factors involved in chondrogenesis. **a** Step-by-step chondrogenesis by growth factors and transcription factors (TFs). Chondroinductive pathways are represented in *green*, while chondroinhibitive signals are depicted in *red*. **b** Activation of intracellular signalling events by growth factor-mediated receptor binding. One growth factor, representative of the chondrogenic phenotype, is TGF-β that promotes type II collagen and GAG production by phosphorylation of SOX9. Remarkably, TGF-β also suppresses hypertrophy through PTHrP activation, which in turn stimulates expression of the TF NKX3.2. BMP-2 is involved in early chondrogenic differentiation by stimulation of mesenchymal condensation. Furthermore, it carries out a chondroinductive effect through hyperacetylation at the Sox9 gene. In later stages, the growth factor is responsible for the initiation of hypertrophy through ALP activation. During in vitro chondrogenic differentiation, on the other hand, BMP-2 is only required for adipose-derived MSCs, while for BM-MSCs, TGF-β is sufficient enough to induce the chondrogenic phenotype. Lastly, IGF-1 plays a role in the differentiation of MSCs into chondroprogenitors through the activation of IGF-RI. Additionally, IGF induces the SOX trio which is commonly known as the mastermind behind functional articular cartilage. *ALP* alkaline phosphatase, *BMP-2* bone morphogenetic protein 2, *HAT* histone acetyltransferase, *IGF-1* insulin-like growth factor 1, *IGF-RI* IGF receptor type I, *NKX3.2* NK3 homeobox 2, *PTHrP* parathyroid hormone-related protein, *RUNX2* runt-related transcription factor 2, *SMAD3* SMAD family member 3, *SMAD4* SMAD family member 4, *SOX9* sex-determining Y region box 9, *TGF-β* transforming growth factor-β. Data based on Bell et al. (1997), Caron et al. (2013), Fischer et al. (2010), Furumatsu et al. (2005), Luyten et al. (1988), Mehlhorn et al. (2007), Osada et al. (1996), Pan et al. (2009), Provot et al. (2006), Wa et al. (2015), Yoon and Lyons (2004), Yoon et al. (2015)

The development of AC takes place during embryonic skeletogenesis. Skeletogenesis begins in the limb bud that is derived from lateral plate mesoderm (Lu et al. 2004). While the lateral plate mesoderm gives rise to the skeleton, connective tissue, and blood vessels, the paraxial mesoderm in the limb develops into muscle precursor cells (Carlson 2013). During early morphological events, pre-cartilaginous condensations appear from the lateral plate mesenchyme in the periphery of the limb bud (DeLise et al. 2000). Following these condensations, a remodelling process is initiated that results in the first sign of joint development marked with flattened cells (Decker 2016). This compact region of flattened cells is defined as the interzone. Interzone-located cells start to express growth and differentiation factor 5 (GDF5) that is mainly responsible for the early development of the joint. Dependent on the following signalling events (i.e. Erg and transforming growth factor-β (TGF-β)), the so-called joint progenitor cells develop into chondrogenic cells that form the articular cartilage (Koyama et al. 2008).While condensations in the periphery of the limb bud give rise to joint-related tissues, the mesenchymal stem cells in central condensations undergo chondrogenic differentiation followed by maturation and proliferation. These cells eventually become hypertrophic chondrocytes that produce type X collagen and matrix metallopeptidase 13 (MMP13) (Bobick et al. 2009). In contrast to articular chondrocytes, hypertrophic chondrocytes maintain a higher proliferation rate and contribute to bone formation via endochondral ossification. Chondrogenesis depends on signalling molecules that act in a temporospatial manner. A schematic representation of the core molecules and chondrogenic signalling events is depicted in Fig. 2. Dependent on the environmental signals that mature articular chondrocytes receive, the cells regulate synthesis of the ECM building blocks: type II, IX and XI collagen, proteoglycans (PGs) and various growth factors and enzymes (Demoor et al. 2014). Type II collagen remains the most abundant isoform that makes up the framework of cartilage (Erggelet and Mandelbaum 2008). A wide range of PGs are embedded in this collagen network and are featured by the unique combinations of glycosaminoglycan (GAG) chains (Gao et al. 2014). Due to the GAG chains, PGs such as aggrecan (ACAN) possess osmotic properties and have the capacity to absorb water which determines the compressive stiffness of the AC (Sophia Fox et al. 2009).

## Cellular reprogramming

In the last decade, cell fate reprogramming through forced TF expression has become a trending research area. Due to the rapid developments, the term ‘reprogramming’ is nowadays used in a broader sense, namely the conversion of cell fate. We therefore distinguish the two approaches as follows: induction of pluripotency is defined as *reprogramming into pluripotency*, whereas *direct reprogramming* is associated with experimentally changing differentiated cell fates bypassing a state of pluripotency.

### Reprogramming into pluripotency

iPSCs were discovered by application of the ‘leave one out’ strategy, also recognized as a top-down approach. The resulting Yamanaka factors essential for pluripotency induction, also known as OSKM—POU class 5 homeobox 1 (Pou5f1 or Oct4), sex determining region Y box 2 (Sox2), Krüppel-like factor 4 (Klf4) and myelocytomatosis oncogene (c-Myc)—were defined from a pool of 24 candidate genes and retrovirally transfected in dermal fibroblasts (DFs). Hereafter, Fbx15-selected iPSCs^1^ were analysed and the authors concluded that the cells were comparable to ESCs in morphology, cell surface markers, gene expression profile and epigenetic state of pluripotency genes (Takahashi et al. 2007; Takahashi and Yamanaka 2006). Nowadays, it is well-accepted that the core TFs—Oct4, Sox2 and Nanog homeobox (Nanog)—are the masters behind pluripotency, pushing the terminal-differentiated cell back up the hill of Waddington’s epigenetic landscape (Waddington 1942; Ladewig et al. 2013; Niwa 2007). Despite the effectiveness of the TF-based approach, many studies observed a low reprogramming efficiency (<4%) (reviewed by Rao and Malik 2012).

According to the stochastic model of Yamanaka (2009), only a fraction of the initial cell population achieves ground-state pluripotency explaining the low efficiency. The model elucidates that the yield of a uniform reprogrammed cell population depends on (1) the TF combination, stoichiometry and concentration and (2) exogenous TF silencing after endogenous TF expression is activated, which in turn depends on (3) the epigenetic signature remarkable for PSCs. Epigenetics plays an important role in cellular reprogramming. In somatic cells, pluripotency genes are repressed by DNA methylation and inhibiting histone markers while fibroblast-specific genes are active. Exposure of these somatic cells to exogenous factors induces changes in epigenetic markers that influence (1) repression of genes associated with the host-specific cell lineage and (2) accessibility of pluripotency genes. However, during this process, epigenetic abnormalities can occur and promote the cells to roll back into their valley due to remaining epigenetic memory of the host cell (Verma and Verma 2011; Sullivan et al. 2010; Kim et al. 2010). Hence, the residual epigenetic signature affects the differentiation potential. Consequently, these iPSCs carry some risks for therapeutic purposes.

### Direct reprogramming

Nowadays, it is well accepted that terminal differentiated cells show a degree of plasticity and are convertible to another cell lineage. Direct reprogramming is comparable with naturally occurring transdifferentiation; however, the dedifferentiation stage is generally not observed in experimental direct reprogramming (Jopling et al. 2011). One of the first cases of direct reprogramming was already demonstrated 20 years ago when Weintraub and colleagues converted fibroblasts into myoblast by transfection of myogenic differentiation 1 (MyoD1) cDNA (Davis et al. 1987). Progress has been made in this field ever since (Akinci et al. 2012; Batta et al. 2014; Ieda et al. 2010; Vierbuchen et al. 2010; Weintraub et al. 1989), and in many of these studies, the key reprogramming factors were developmental regulators of the target cell lineage. Although direct reprogramming holds great promise for regenerative medicine, the mechanism of action is cell type-specific and still largely unknown for many cell types. It is suggested that cells are converted through down-regulation of the original cell-specific genes, while simultaneously activating target cell-specific genes (Sancho-Martinez et al. 2012). Another model proposes the involvement of a dedifferentiation step before the cells are redifferentiated into another cell type. However, many scientists failed to observe the latter mechanism.

Similar to reprogramming into pluripotency, low efficiency affects the direct reprogramming approach. Yet again, this is due to the epigenome of the original cell that impedes appropriate reprogramming by exogenous TFs (Chin 2014; Sancho-Martinez et al. 2012).

### Reprogramming into chondrocytes

Research into cartilage regeneration by iPSCs or direct conversion is still in early phase. Since ESCs were the first pluripotent stem cells closely examined for chondrogenic capabilities, these differentiation strategies (Nakagawa et al. 2010; Oldershaw 2012; Han et al. 2010) have also been applied to iPSCs. Many studies aim to optimize the approach by mimicking in vivo chondrogenesis. However, it has not yet been proven that ESCs and iPSCs share the same mechanism of action during chondrogenic differentiation. Furthermore, it is recommended to perform in vitro chondrogenic differentiation through 3D cell culture models in order to prevent dedifferentiation or hypertrophy of the induced chondrocytes.

In contrast to iPSC-mediated chondrocyte generation, direct conversion of fibroblasts into chondrocytes has not been extensively explored (Hiramatsu et al. 2011; Outani et al. 2013). Most likely, this process also takes place in a 3D environment to prevent dedifferentiation. Moreover, it remains important to generate functional and stable chondrocytes with TF-based reprogramming techniques to, in the future, implement these approaches in clinical practice. In order to accomplish this milestone in cartilage regeneration, the chondrocytes need to mimic native articular chondrocytes as closely as possible.

The quality of the reprogramming method for clinical application depends on two factors: (1) the time frame wherein the starting cells are converted into the desired cell type and (2) the efficiency of this conversion. In general, the time frame is determined by the TF combination and the culture conditions. The reprogramming efficiency functions as an exact measurement. In order to assess the overall efficiency, two parameters have to be evaluated: (i) the yield, defined as the percentage of converted cells relative to the number of starting cells, and (ii) the purity, which accounts for the percentage of functional converted cells in the final cell population. Preferably, the costs are also taken into account to assess the cost-effectiveness and cost-efficiency of the method. However, precise cost measurements are difficult and, therefore, only the reprogramming efficiency and the time frame are included to examine the quality of the reprogramming method.

The quality of the generated chondrocytes depends on several criteria that underline the safety and functionality of the cells. Firstly, the cell identity is important to assess to what extent the converted cells adopted a phenotype and genotype similar to their native counterparts. Since articular chondrocytes do not possess a cell surface marker, the chondrogenic identity is confirmed when the following markers are expressed and produced: type II collagen, ACAN, GAG and SOX9. Additionally, a polygonal morphology characterizes the chondrogenic phenotype. Secondly, since clinical application is the purpose, the safety of the converted cells has to be validated on the basis of (1) genomic stability and (2) epigenetic assembly. Both parameters are closely associated with the tumourigenic features of the converted cells. Karyotyping, whole genome and epigenome sequencing and teratoma analysis are the most common assays to study safety. Preferably, transgenes are silenced or even absent in the final cell population meaning that screening of pluripotency factors (e.g. Oct4, Sox, Nanog) is also required.

The most relevant criterion for cartilage regeneration is the functionality of the derived chondrocyte-like cells both in vitro and in vivo. Evidently, the converted cells have to acquire the capacity to produce and distribute cartilage-specific ECM, eventually leading to the formation of hyaline cartilaginous tissue. However, there is a risk of hypertrophic activity that affects the cartilage-like tissue and results in endochondral ossification. Furthermore, residual epigenetic memory of the starting cell population might affect cartilage-specific ECM architecture by the production of fibroblastic type I collagen. For these reasons, both hypertrophic (i.e. type X collagen, MMP13) and fibroblastic markers have to be assessed in order to gain insights into the cells’ functionality. Cell survival, integration in native tissue and susceptibility to physiological stimuli also define the functionality of the cells.

## The iPSC-based detour

### From fibroblast to pluripotency

Numerous advanced TF delivery methods to induce pluripotency have been introduced. The method is particularly relevant since it influences the reprogramming efficiency and quality of the iPSC population (Alateeq et al. 2015). Although no consensus has been reached regarding the definition of iPSC quality, they are characterized by their ability to act similar to ESCs. From a genetic perspective, this means that the cells’ genome does not show alterations dissimilar from the starting cell pool (safety). Thus, the absence of transgenes and DNA damage needs to be guaranteed to prevent tumourigenic characteristics. When cells adhere to these aspects, they are labelled with a ‘high-quality mark’. How these features are examined varies in the academic literature.

Subsequently, it is widely acknowledged that non-integrative transduction methods are preferred since these initiate footprint-free iPSCs. Belonging to this group are integration-free viral vectors such as adeno- and Sendai viral viruses (Fusaki et al. 2009; Zhou and Freed 2009), episomal plasmids (Okita et al. 2011), mini-circles (Jia et al. 2010), messenger RNA (mRNA) transfection (Luni et al. 2016) and protein delivery systems (Kim et al. 2009). A state-of-the-art reprogramming method involves microfluidic environments with daily managed delivery of modified mRNA (mmRNA) (Luni et al. 2016). Noteworthy is that all methods differ in efficiency, quality, costs and labour intensity. Nevertheless, advancements in cell culture technologies and reprogramming methods provide opportunities for clinical translation. A final consideration regarding the method of interest is the intellectual property landscape that may affect actual implementation.

While integrative delivery systems result in high efficiencies with low iPSC quality, non-integrative reprogramming methods are characterized with remarkably low efficiencies and clinical-grade iPSC generation. Improvement of reprogramming efficiency depends on the identity, interplay and concentration of exogenous TFs, which are in turn related to the starting cell’s genome. In this paper, fibroblasts found the basis for iPSC reprogramming.

#### Transcription factor identity, combination and concentration

Reprogramming fibroblasts into iPSCs is mainly accomplished by transfection with the original OSKM factors. Since c-Myc is an oncogene, many scientists aim to replace or eliminate this TF in order to prevent tumourigenic characteristics (Han et al. 2010; Chen et al. 2011; Li et al. 2011a; Maekawa et al. 2011). C-Myc has been replaced by factors such as L-Myc, transforming growth factor-β (TGF-β) inhibitors and ascorbic acid/Vitamin C (VitC) and have shown decreased tumourigenesis and enhanced efficiency (Han et al. 2010; Chen et al. 2011; Li et al. 2011a; Maekawa et al. 2011). During reprogramming, a transition to an epithelial cell state is required and accelerates the reprogramming process. This mesenchymal-to-epithelial transition (MET) of fibroblasts is prevented by TGF-β-activity (Li et al. 2010). Normally, c-Myc is enough to suppress both TGF-β1 and TGF-βR2. By replacing c-Myc with TGF-β inhibitors, the oncogene’s function is taken over by a likely clinical more appropriate alternative. Others have proven that c-Myc is dispensable, although the process resulted in a lower induction of pluripotency that led to latency (Nakagawa et al. 2010; Wernig et al. 2008).

In addition to factor identity and combination, the concentration influences reprogramming efficiency since TFs can interact with chromatin- and histone-modifying proteins to remove barriers (Cota et al. 2013; Hanna et al. 2009; Yamanaka 2009). It is considered that a higher amount of TFs accelerates the actions of the remodelling proteins and enhances reprogramming efficiency.

#### Small molecules and chemical compounds

**Fig. 3:**
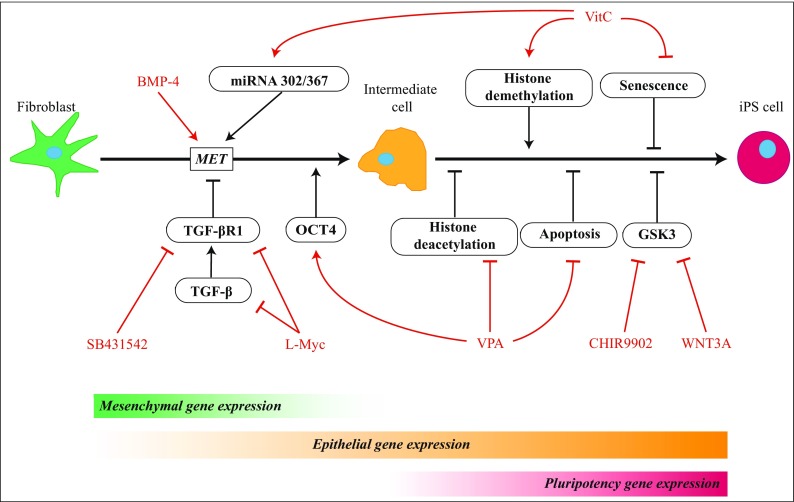
Improvement of the reprogramming process and replacement of c-Myc by small molecules and chemical compounds. c-Myc is normally involved in facilitation of MET via the transforming growth factor-β (TGF-β) pathway. As shown, the oncogene can be replaced by SB431542 and L-Myc, an Myc family member with lower transformation activity. Furthermore, the positive effects of valproic acid (VPA), vitamin C (VitC), wingless type-3a (WNT3A) and CHIR9902, a GSK3 inhibitor, on iPSC generation are depicted, as well as bone morphogenetic protein 4 (BMP-4) and its function as MET inducer. VPA improves cell proliferation and inhibits apoptosis through the suppression of the p16/p21 pathway (Zhai et al. 2015). Also, VPA directly activates the Oct4 promoter through stimulation of the PI3K/Akt/mTOR signalling pathway (Teng et al. 2014). VitC acts mainly through histone demethylases that reduce H3K36 methylation patterns. In addition, VitC-activated Jhdm1b has shown to cooperate with OCT4 in order to activate the miRNA 302/367 cluster which in PSCs is a downstream target of OCT4 (Barroso-delJesus et al. 2008) and increases reprogramming efficiency by promoting MET (Subramanyam et al. 2011)

Besides TFs, other reprogramming factors such as small molecules and chemical compounds are widely examined to replace TFs or boost the reprogramming approach (Nie et al. 2012; Bar-Nur et al. 2014). These molecules act in different manners yet are all focussed on lowering the epigenetic or signalling barriers that hamper proper and efficient reprogramming (Fig. 3). In other words, the small molecules are able to modulate endogenous protein levels and cellular signalling events that enhance iPSC generation. Examples include a facilitated MET, a metabolic switch to glycolysis and epigenetic modification of DNA and histones to a more ‘open’ state (Su et al. 2013). To highlight the latter, PSCs are characterized with highly acetylated genes (open/active), while somatic cells are marked with highly methylated genes (closed/inactive). Hence, inhibition of enzymes responsible for methylation of DNA and histones (i.e. histone deacetylase and DNA methyltransferase) (Greer and Shi 2012) steers the cells towards an open genome which benefits the transition to PSC.

Valproic acid (VPA) is a common histone deacetylase (HDAC) inhibitor that has proven to accelerate reprogramming in combination with exogenous OCT4 and SOX2 expression (Huangfu et al. 2008). One-month treatment of DFs with 0.5–1 mM VPA already yielded a 1000-fold increase in efficiency and cell colonies that resembled the gene expression profiles of ESCs. The underlying mechanism of VPA has not yet been clarified. Since VPA-regulated HDAC inhibition is non-specific, it is likely that the molecule stimulates euchromatin structure in general.

VitC was initially introduced as an antioxidant in order to reduce reactive oxygen species (ROS) caused by OSK minus c-Myc reprogramming (Esteban et al. 2010). Down-regulation of ROS prevents cell senescence, and cells thus become more amendable to reprogramming. Noteworthy is the combination of VitC and VPA that further enhances the reprogramming efficiency, suggesting a synergistic effect. Lastly, research has shown VitC-dependent epigenetic modifying effects in mouse fibroblasts through Jhdm1a/1b activity, confirming that epigenome remodelling accelerates the reprogramming process (Esteban et al. 2010; Wang et al. 2011).

Finally, CHIR99021 has the potential to initiate expression of pluripotency genes by inhibition of glycogen synthase kinase 3 (GSK3) that in turn allows β-catenin to move into the nucleus where this transcription factor initiates expression of OCT4 and NANOG (Han and Yoon 2011). Notably, in combination with VPA and minimally required TFs (i.e. only Oct4), reprogramming efficiency is improved to 0.3%, thus making Sox2 and Klf4 dispensable (Li et al. 2011b).

Above elaborated small molecules have proven to be powerful alternatives or supplements for the reprogramming factor by lowering major epigenetic and signalling barriers. Nevertheless, there are limitations to small molecule-only reprogramming approaches such as non-specific activity and increased costs due to continuous treatment.

### From pluripotency to chondrocyte

**Fig. 4:**
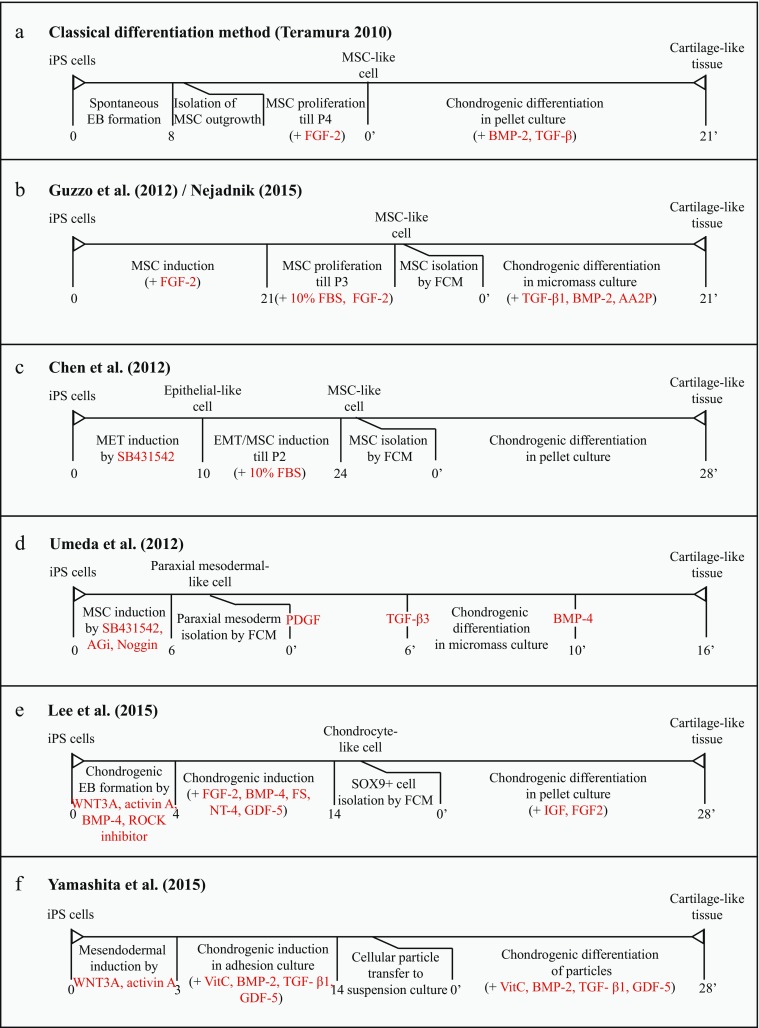
Schematic representation of the chondrogenic culture protocols described in chapter 3.2. *AA2P* ascorbic acid 2-phosphate, *AGi* glycogen synthase kinase-3 (GSK3) inhibitor, *BMP-2* bone morphogenetic protein 2, *BMP-4* bone morphogenetic protein 4, *FBS* foetal bovine serum, *FCM* flow cytometry, *FGF* fibroblast growth factor, *GDF-5* growth and differentiation factor 5, *IGF* insulin growth factor, *NT-4* neurotrophin 4, *PDGF* platelet-derived growth factor, *SB431542* TGF-β inhibitor, *TGF-β* transforming growth factor-β, *VitC* vitamin C/ascorbic acid, *WNT3A* wingless type family member 3a

Traditionally, chondrogenic differentiation is carried out through spontaneous embryoid body (EB) formation by iPSCs (Fig. 4a) (Teramura et al. 2010). However, we concentrate on multistep-directed differentiation methods since this type of approaches matches in vivo chondrogenic differentiation of PSCs more closely and is often described in academic literature.

The most simple directed culture method involves initiation of MSC-like cells followed by growth factor-induced chondrocyte differentiation. Guzzo et al. (2013) demonstrated the development of MSC-like cells after undifferentiated human induced pluripotent stem cells (hiPSCs) were directly seeded on gelatin-coated plates and stimulated by MSC induction media (Fig. 4b). The derived hiPSC-MSC-like cells showed substantial matrix production and expressed SOX9, COL2A1 and ACAN, while collagen type I alpha 1 (COL1A1) and COLXA1 remained minimally expressed. Interestingly, the iPSC-MSC-like cells showed a higher chondrogenic potential compared to bone marrow-derived MSCs; expression of the SOX trio was significantly higher, whereas runt-related transcription factor 2 (RUNX2) and COL1A1 expression was low in iPSC-MSC-like cells. These data indicate that iPSC-derived MSCs offer advantages over BM-MSCs (Guzzo et al. 2013). Nejadnik et al. (2015), who followed a comparable protocol, observed a conversion of 90% of the hiPSCs to hiPSC-MSC phenotype, which is highly efficient compared to EB-regulated MSC generation. The hiPSC-MSC-chondrocytes established by pellet culture and encapsulation in PEG/CS hydrogels expressed cartilage markers. Meanwhile, immunostaining revealed the presence of COL10A1 that ideally should be low.

There are multiple studies that use standard MSC induction medium and culture for approximately 2–3 weeks before a uniform MSC population is visible (Hynes et al. 2013; Kang et al. 2015). A way to improve this process is given by Chen et al. (2012) who stimulated chronologically MET and epithelial-to-mesenchymal transition (EMT) by addition of small molecules (i.e. TGF-β pathway inhibitor SB431542) (Fig. 4c). Consequently, the cells adapt an epithelial phenotype and MSC markers are barely expressed. However, when the cells are transferred to MSC induction medium, up-regulation of MSC cell surface markers could already be detected after one to two passages. Furthermore, the iPSC-MSC-like cells possessed trilineage differentiation capacity, had a normal karyotype and did not form teratomas, thereby being clinically safe and functional. Umeda et al. (2012) further specified the method by administration of a GSK3 inhibitor, Noggin and SB431542 (Fig. 4d). Subsequently, chondrogenic paraxial mesoderm appeared from iPSCs that underwent sequential chondrogenic differentiation using platelet-derived growth factor (PDGF), TGF-β3 and bone morphogenetic protein (BMP)-4. This approach further fragmented chondrogenesis, which gave rise to more hyaline-like cartilaginous particles compared to MSC-derived cartilage-like constructs. Remarkably, activin A could induce paraxial mesoderm from ESCs, whereas it does not have this capacity in iPSCs. This indicates that iPSC and ESCs differ in growth factor susceptibility and adopt distinct differentiation pathways.

Beside the three-stage chondrocyte differentiation methods, many reviews and articles cite the multistage protocol from Oldershaw et al. (2010), which sequentially exposed the cells to different growth factors in combination with substrates of matrix proteins. The protocol, which exploits feeder-free and serum-free culture media, produced ESC-derived chondrocyte-like cells with high expression of Col2A1 and Sox9 and sulphated GAG deposition. Significant growth factors include wingless-type family member 3a (WNT3A), activin A, follistatin (FS), BMP-4, FGF-2 and growth and differentiation factor 5 (GDF-5). While the protocol was designed for PSCs, multiple experiments on iPSCs were unsuccessful wherein decline in iPSC viability was the main limitation. Optimization of the stage-gating method was focussed on the modulation of Wnt- and TGF-β-signalling that could improve the generation of chondrogenic mesoderm (Umeda et al. 2012). Lee et al. (2015) introduced the short-term differentiation of hiPSCs in EBs with chondrogenic mesodermal preferences (Fig. 4e). Along with this, the p160-ROCK inhibitor Y27632 was added to prevent dissociation-induced iPSC apoptosis. Beside this function, ROCK inhibition is associated with increased GAG synthesis and elevated SOX9 expression in differentiated chondrocytes (Woods et al. 2005), making this small molecule also beneficial for chondrogenic differentiation. This protocol yielded 98% SOX9^+^ cells. Pellet cultures resulted in expression levels of the SOX trio as well as COL2A1 and ACAN comparable to primary adult chondrocytes. Interestingly, cell proliferation was enhanced and induced chondrocytes continued to express SOX9 after 14 passages. This proliferation capacity is remarkable for chondrocytes and could be beneficial for therapeutic applications. Finally, safety and functionality were assured by the absence of subcutaneous teratoma formation and the presence of cartilage-like tissue similar to the control adult chondrocyte-laden hydrogels. The authors suggest that the factors involved in the RhoA/ROCK-, Wnt- and activin A signalling pathways led to increased chondrogenic differentiation of the hiPSCs.

While most studies require a 3D cell culture assay to effectively engineer cartilage construct, Yamashita et al. (2015) recently introduced a strategy to generate scaffoldless hyaline cartilaginous tissue construct from hiPSCs (Fig. 4f). Briefly, hiPSCs-MSCs were exposed to VitC, BMP-2, TGF-β1 and GDF-5 (abbreviated as ABTG). Remarkably, at day 42 the chondrogenic particles showed increased type I collagen staining and maintained this expression up to day 140. The replacement of chondrogenic medium at day 42 with conventional medium resulted in increased GAG staining, while type I collagen expression was decreased. These results suggest that ABTG is only necessary for a specific culture period, in which hiPSCs are committed to the chondrogenic lineage, but it is not continuously required. Uniquely, this protocol exhibits purification steps without the use of cell sorting techniques. The researchers namely suggest that non-mesendodermal cells undergo apoptosis due to the low foetal bovine serum (FBS) concentrations. Furthermore, transfer of non-adhesive nodules to a suspension culture contributed to the removal of non-chondrogenic cells, thus resulting in a pure chondrogenic cell population.

In conclusion, it is of great importance to apply the most optimal set and spatiotemporal pattern of growth factors to steer the iPSCs chronologically towards the chondrogenic cell lineage. According to the available studies, the main growth factors applied to induce iPSC towards MSC-like cells are WNT3A, activin A and FGF-2 or small molecules that interfere with the Wnt- and TGF-β-pathways. Further differentiation of (chondrogenic-committed) MSC-like cells is established by exposure to common chondroinductive growth factors, such as TGF-β homologues, BMP-2/4 and GDF-5. These molecules are consistent with the compounds identified by Yang et al. (2012) that were shown to promote chondrogenesis in iPSCs. Secondly, inclusion of cell selection and isolation techniques is necessary to increase the yield and safety of the resulting chondrogenic cell population. Unfortunately, chondrocyte-specific surface markers have not been identified to date, precluding flow cytometry (FCM). However, the selection of chondrocyte-like cells can be accomplished with cartilage-specific gene-GFP constructs such as Col11A2-EGFP reporter genes (Diekman et al. 2012).

## Direct reprogramming into chondrocytes

### Intra-mesodermal reprogramming

Within the mesoderm, studies have shown the ability to directly convert fibroblasts into functional myocytes (Weintraub et al. 1989), cardiac muscle cells (Efe et al. 2011; Ieda et al. 2010), macrophages (Feng et al. 2008) and chondrocytes (Hiramatsu et al. 2011). Many reports discuss the concept that cells with shared epigenetic landscapes will reprogram more efficiently. This may be explained by the shared cell progeny and the degree of similarity between epigenetic marks and profile. Basically, this mutual epigenetic profile indicates that less epigenetic barriers have to be crossed (Sebban and Buganim 2016; Bernstein et al. 2007). Indeed, in Waddington’s epigenetic landscape, cell lineages within a germ layer are separated by lower ‘hills’ compared to the hills between different germ layer-originating cell types (Ladewig et al. 2013). However, direct lineage reprogramming and thus avoidance of a pluripotent state may result in preservation of host cell-originating epigenetic barriers that might affect the stability of the induced cell fate. As a consequence, the cells ‘roll back’ to the starting cell lineage.

#### Mechanism of action

Similar to iPSCs, fibroblasts are exposed to the top-down approach that eventually results in a core TF cluster for the desired cell lineage. Since this method is cost-consuming, inefficient and unscalable, a predicate computational system (Mogrify) provides an alternative for identification and validation of reprogramming factors (Rackham et al. 2016). Intra-mesodermal reprogramming is mainly demonstrated by administration of lineage-instructive TFs to fibroblasts, in this article referred to as lineage-instructive reprogramming. During this approach, it is assumed that epigenetic barriers cross-over without passing a pluripotent cell fate. Alternatively, an epigenetic activation phase can be introduced (Pournasr et al. 2011) before lineage-specific development clues are applied. This approach involves transient low-level expression of Oct4, Sox2, Klf4 and c-Myc that stimulates a partially pluripotent intermediate stage (Efe et al. 2011). According to this study, efficiency is improved by several folds, suggesting that a more open chromatin state enhances susceptibility to lineage-specific factors and subsequently accelerates the reprogramming process. Indeed, the pluripotency factors induced removal of epigenetic barriers resulting in a dedifferentiation or progenitor intermediate cell state (Ang et al. 2011; Pournasr et al. 2011; Ma et al. 2013b). Following this, lineage-instructive TFs stimulate the differentiation into the desired somatic cell, which is associated with the adoption of a ‘new’ epigenetic regulatory network.

Theoretically, this method provides a short time frame that allows cell expansion due to self-renewal capacities of the cells. For cartilage regeneration, this would be beneficial since clinical implantation requires a high density of chondrocytes. However, full acquisition of a pluripotent state has to be prevented since prolonged induction of the pluripotency network has shown to inhibit cell type conversion (Efe et al. 2011).

### From fibroblast to chondrocyte

Although DFs are readily accessible and show distinct expansion capacities, the propensity to produce high levels of type I collagen is an obstacle. Residual fibroblastic cell features may affect in vivo regeneration of AC and instead result in fibrocartilaginous tissue. Another concern is the risk of hypertrophic activities of induced chondrocytic cells, which is often observed in chondrogenic differentiation of BM-MSCs (Pelttari et al. 2008). Nevertheless, prior studies have shown the ability of DFs to differentiate into the chondrogenic phenotype and the formation of cartilage-mimicking tissue from these cells, simply by modification of culture conditions (Cui et al. 2004; Junker et al. 2010; Mizuno and Glowacki 1996; Yates et al. 2004).

#### TF-mediated reprogramming

Hiramatsu et al. (2011) were the first who demonstrated chondrocyte generation of fibroblasts by forced TF expression. Continuous retroviral expression of c-Myc, Klf4 and Sox9 (which have shown to be indispensable) yielded colonies with an efficiency of 0.3%. However, after polygonal morphology assessment and GAG staining, the efficiency decreased to 0.08%. Notwithstanding, positive GAG, type II collagen and ACAN were observed after pellets were cultured with chondrogenic-induced mouse DFs (iChon). On the contrary, hypertrophic gene expression (i.e. type X collagen and Mmp13) was undetectable. Interestingly, the authors included DNA methylation analysis to assess the differences in promoter accessibility. The iChons contained methylated CpGs in the promoters of fibroblast-associated genes Col1a1 and Col1a2, while in the parental MDFs these promoters were unmethylated. This indicates that epigenetic reprogramming took place. Controversially, two out of seven induced cell lines showed significant levels of type I collagen expression. However, these expression levels might be explainable by a pool of incompletely converted fibroblasts. Unfortunately, the iChons appeared to be unsafe since inappropriate karyotyping and subcutaneous tumour formation with abundant type I collagen were observed. With persistent c-Myc expression in mind, the researchers switched to transient TF expression with a DOX-inducible lentivirus. Cultured in chondrogenic induction medium (containing 1% FBS, TGF-β1, GDF-5 and AA2P), the cells maintained high endogenous Sox9 expression. Nevertheless, close examination demonstrated elaborated expression of Col10a1 and Mmp13. This is consistent with the observations of Tam et al. (2014) who compared constitutive (iChon^con^) and transient (iChon^ind^) chondrogenic-induced MDFs regarding cellular hypertrophy and stability. Upon chondrogenic differentiation, both iChon^ind^ and iChon^con^ showed significant expression of type II collagen, while type X collagen was only increased in iChon^ind^. Similarly, in vivo analysis associated iChon^ind^-regulated tissue formation with bone-like characteristics, whereas iChon^con^ were able to form stable cartilage containing cells. In addition to the results of Hiramatsu et al., this suggests that prolonged exogenous Sox9 expression is required to obtain chondrogenic cells resistant to hypertrophy. It is surprising that high endogenous Sox9 expression, present in transient-induced iChons, is not able to inhibit these hypertrophic characteristics while Sox9 is known to inhibit osteochondral ossification of native chondrocytes (Hattori et al. 2010; Liao et al. 2014).

A follow-up study performed in human DFs (hDFs) exhibited remarkable differences with mDFs (Outani et al. 2013). The hiChons for example did not produce type I collagen after a sustained period of pellet culture. Additionally, COL10A1 and MMP13 gene expression was undetectable and subcutaneous injection of the cells did not lead to tumour formation, but cartilage-like nodules did. Also, hDFs were more susceptible to the reprogramming approach with a conversion efficiency of 0.24%. In general, the above elaborated reprogramming approach does not involve a pluripotent intermediate state since mRNA expression of PSC markers was not detected (Outani et al. 2011). However, a progenitor intermediate state recognized by dedifferentiation marks cannot be excluded by the evidence presented.

Overall, the choice between constitutive and transient direct lineage reprogramming into chondrocytes remains a topic that has to be further explored. Ideally, transgene integration is avoided in order to prevent teratoma formation, increasing the suitability for clinical application (Sebban and Buganim 2016). Nevertheless, it is acknowledged that continuous expression of the reprogramming factors c-Myc, Klf4 and especially Sox9 is required.

#### Non-TF-mediated reprogramming

As already mentioned, direct reprogramming into chondrocytes was demonstrated by modified culture conditions, hence without exogenous TF expression. In 1996, Mizuno and Glowacki showed chondrogenic effects of demineralized bone powder on DFs cultured in 3D compositions. Nowadays, several studies identified agents, biomaterials and scaffolds that facilitate direct reprogramming towards chondrocyte-like cells (Bussmann et al. 2013; Kino-Oka et al. 2009; Nam et al. 2014; Yin et al. 2010). Medium containing low FBS concentration (2–5%) and GDF-5 is sufficient to induce chondrogenic characteristics, such as endogenous SOX, ACAN and type II collagen expression, by DFs in micromass culture (Yin et al. 2010). Despite the simple protocol, high type I collagen expression is sustained, assuming that the original epigenetic program is not completely reset and/or non-chondrogenically differentiated DFs are still present. Exposure of hDFs to 5% FBS, TGF-β3 and AA2P embedded in a soft hydrogel (i.e. RAD16-I peptide network) improved chondrogenic differentiation of hDFs (Bussmann et al. 2013). Although enhanced and homogeneous cartilage-specific ECM production was observed, type I collagen remained present during the whole culture period. However, a low level of type I collagen is usually also found in native AC. Interestingly, in vitro passaged chondrocytes and fibroblasts share phenotypic characteristics (Nam et al. 2014; Kino-Oka et al. 2009). Also, dedifferentiation of chondrocytes in monolayer expansion is correlated with increased expression of fibre-associated collagens (Goessler et al. 2004). Counteraction by redifferentiation approaches in 3D models (Schulze-Tanzil et al. 2002; Das et al. 2013) might correspond to the chondrogenic conversion of fibroblasts and improve the quality of iChons. This way of reasoning indicates that epigenetic similarities between fibroblasts and chondrocytes co-exist.

Another culture modification widely studied in MSC-based chondrogenic differentiation is the introduction of a hypoxic environment. In brief, low oxygen increases the production of hypoxia-inducible factor 1α (HIF-1α) that in turn initiates transcription of genes such as type II collagen, Sox9 and chondroitin sulphate (Kanichai et al. 2008; Robins et al. 2005). Hypoxia and HIF-1α are also acknowledged to simultaneously inhibit expression of COL1A1 and COL1A2 (Duval et al. 2009). Therefore, it is hypothesized that low oxygen concentrations (5% O_2_) improve chondrogenic conversion of fibroblasts and regulates fibroblastic type I collagen production. Indeed, fibroblasts cultured in hypoxic conditions and chondrogenic medium have shown to lower type I collagen abundance compared to normal culture conditions (20% O_2_) (Kalpakci et al. 2014; Singh et al. 2011). Although hypoxic differentiation advances the cells further towards a chondrogenic phenotype, the attendance of residual fibroblasts cannot be excluded and therefore represents a risk for human transplantation.

To conclude this chapter, DFs, especially from human origin, are highly susceptible and amendable to direct lineage reprogramming. Groundbreaking studies in this field were presented by Hiramatsu and Outani who demonstrated the expression of cartilage-specific markers. Also, proliferation activities were high and therefore beneficial for cartilage regeneration. Reprogramming efficiencies were rather modest and comparable with the iPSC detour. Limitations to overcome include hypertrophic chondrocyte maturation and the deposition of abundant type I collagen. The latter may be caused by an incomplete epigenetic reset that results in partially converted cells. Hypertrophy can only be prevented by constitutive exogenous TF expression; however, integrative reprogramming methods are unfavourable for clinical translation. Lastly, a pluripotent state was negligible. However, the involvement of a progenitor state is not excluded and might even be valuable. Moreover, epigenetic similarities between the cell types may enable direct reprogramming. Further research into the epigenetic progeny of the two cell lineages may offer valuable insights for the approach to eventually generate long-term functional and stable chondrocytes safely and efficiently.

## Discussion

In this paper, two distinct reprogramming approaches are examined as a potential strategy to obtain a new cell source for AC regeneration. From a clinical perspective, the derived chondrocyte population has to be safe, functional and sufficient in size. As demonstrated in the chapters, the quality of the chondrocyte-like cells highly depends on the type of reprogramming method including the combination and concentration of TFs and the manner of exposure (i.e. constitutive or transient exogenous expression). In addition to forced TF expression, both reprogramming methods may be improved by small molecules, chemical compounds and functionalized 3D cell culture systems. An overview of several aspects with regard to the reprogramming strategies and resulting chondrogenic cells is given in Table 1.

**Table 1:** An overview of the two reprogramming methods and the quality of the derived chondrocytes

	iPSC-based ‘detour’		Direct lineage reprogramming
	*Fibroblast to iPSC*	*iPSC to chondrocytes*	*Fibroblast to chondrocyte*
Efficiency	<4%	89–97%	<0.24%
Time frame	Weeks–month	3–8 weeks	Hours–days
c-Myc requirement	No^a^	N.A.	Yes
Transgene dependency	No	No	Yes
Transgene silencing required	Yes	N.A.	No
Expandable	Yes	Yes	Yes^c^/no^d^
Risk for tumourigenesis	N.A.	Yes, low^b^	High^c^/low^d^
Fibroblast markers	Yes, very low	N.I.	Yes, moderate
Hypertrophic activity	N.A.	Low	Low^c^/high^d^

*N.A.* not applicable, *N.I.* not included

^a^c-Myc can be replaced by L-Myc, TGF-β inhibitors and vitamin C

^b^As long as isolation and purification of the intermediate cell population are included

^c^Constitutive

^d^Transient

Cartilage formation through iPSC generation is a two-stage approach that is acknowledged as a protracted and inefficient method. Nevertheless, the strategy is suitable for the acquisition of a sufficient number of chondrocytes due to enhanced self-renewal of iPSCs. Active cell proliferation is favourable since it ‘opens’ the starting cell’s genome and allows rapid modifications towards pluripotency. Originally, entrance of the cell cycle is initiated by c-Myc. However, the risk for random mutagenesis that reduces the safety of the iPSCs has to be limited for clinical application. Therefore, substitutes such as L-Myc, TGF-β inhibitors and VitC provide alternatives to the oncogenic gene. Noteworthy is that the initiation of MET with TGF-β inhibitors improves the initial stage of reprogramming into pluripotency. For this reason, it is suggested that the generation of iPSCs requires a transition to an epithelial gene expression pattern and that epithelial cells might be more efficient. Indeed, skin keratinocytes have shown a more efficient and rapid iPSC generation compared to human fibroblasts (Aasen et al. 2008). Interestingly, Borestrom et al. (2014) created chondrocyte-derived iPSC with a higher matrix-producing capacity compared to fibroblast-derived iPSCs after chondrogenic differentiation. Thereby, the researchers show that the starting cell type for the iPSC detour towards clinical cartilage influences the quality of derived chondrocytes. Future studies into cartilage formation through iPSC generation need to consider the type of starting cell and the effect on resulting chondrocytes.

Most researchers performed directed chondrogenic differentiation by temporal and sequential application of defined factors in order to mimic embryonic chondrogenesis. This stage gating provides advantages over other methods in a way that it produces a high cell yield and a homogeneous chondrocyte population. However, cell homogeneity highly depends on the inclusion of appropriate (intermediate) cell selection techniques. The cell yield is difficult to assess since directed differentiation induces high proliferation rates. Thus, the effect of temporal growth factor exposure on differentiation efficiency remains unclear. Interestingly, the iPSC-derived MSC-like cells show higher expansion capacity and reduced chondrocyte maturation in contrast to native adult MSCs. From this point of view, iPSCs provide more profits over MSCs as an adult stem cell source for cartilage regeneration.

Cell yield and homogeneity as well as culture period can be further improved by early initiation of paraxial mesodermal cells. Remarkably, paraxial mesoderm differentiation from iPSCs is characterized by the same molecular factors involved in iPSC generation from fibroblasts, including Wnt3a, activin A, SB431245 and BMP-4. Differential effects of these components might be explainable by the difference in starting cell type (iPSC and fibroblast, respectively). In PSCs, it has indeed been shown that the TGF-β/activin/nodal branch of the TGF-β signalling pathway plays a crucial role in the maintenance of stem cell identity (James et al. 2005). Thus, when TGF-β is inhibited by SB431245, the PSCs enter the early cell fate determination phase while in later stages of differentiation TGF-β activity enhances chondrocyte development (Yang et al. 2009). Thus, exposure of iPSCs to factors such as TGF-β has to be performed in a critical time- and dose-dependent manner to prevent incorrect cell fate determination. Nowadays, many researchers examine in vitro directed chondrogenesis. However, a distinction between ESC-, iPSC- and adult MSC-mediated chondrocyte differentiation has to be kept in mind.

A serious drawback of iPSC-based chondrocyte development is the prolonged culture period (approximately 2–3 months) and the high costs of all the transcription and growth factors, extracellular matrix molecules and 3D niches necessary for efficient iPSC generation and especially directed chondrogenic differentiation. Time and costs may be diminished by the use of allogeneic clinical-grade iPS cell lines that match the patient’s human leukocyte antigen (HLA) type (Turner et al. 2013). Indeed, efforts are initiated in iPSC banking to offer high-quality allogeneic iPSCs cultured under GMP conditions (Taylor and Jones 1979; Turner et al. 2013). These donor banks may facilitate regulatory approval due to extensive donor screening and testing compared to autologous iPSC generation. Also, the implantation of allogeneic cells into cartilage defects may be allowed more easily since AC is immuno-privileged due to its avascular nature.

In contrast to the iPSC detour, intra-mesodermal direct lineage reprogramming may require less epigenetic barriers to be crossed due to a mutual epigenetic landscape. Therefore, a lower epigenome remodelling rate is needed and this may reduce the risk of random mutagenesis and hence teratoma formation. Indeed, fibroblast-derived chondrocytes did not develop tumours in immunodeficient mice which may be explained by the shortened culture period with c-Myc. Opposite to teratoma formation, it is serious to consider the risk for fibrocartilaginous cartilage. Since chondrocytes cannot be isolated from fibroblasts on the basis of cell surface markers, there remains a high risk on the presence of non- or partially converted fibroblasts that in vivo contribute to the formation of non-functional fibrocartilaginous cartilage.

A serious issue of existing direct conversion techniques is the requirement of constitutive transgene expression. In particular, exogenous Sox9 seems to be indispensable to generate stable cartilaginous tissue and prevent hypertrophy. Meanwhile, high endogenous levels of Sox9 were not able to suppress hypertrophic maturation, suggesting that the activity of endogenous Sox9 was impaired. Posttranslational modifications of Sox9 via PKA-mediated phosphorylation determine the activity of Sox9 and to what extent the protein inhibits hypertrophy (Akiyama 2008). Also, the parathyroid hormone-related protein (PTHrP) receptor is mainly responsible for Sox9 phosphorylation (de Crombrugghe et al. 2000). It might be possible that this cartilage-specific receptor is not yet activated and alters Sox9 activity. On the other hand, it has been suggested that the epigenetic status of the cell influences the Sox9-regulated transcriptional network for chondrocyte differentiation (Furumatsu and Asahara 2010). According to Leung et al. (2011), Sox9 inhibits Col10a1 transcription by binding to the gene’s regulatory elements. Thus, it is argued that this regulatory domain is not yet accessible for Sox9 in chondrogenic-induced fibroblasts. Finally, Takahashi (2012) proposed that the endogenous gene products act in synergy with the exogenous factors to enable activation of the full chondrogenic transcriptional network. Hiramatsu and colleagues stimulated the cells for only 2 days, which suggests that prolonged exogenous transduction is mandatory to completely activate the chondrogenic transcription circuit in fibroblasts. In addition to this, the researchers cultured the induced cells in monolayer, though it is commonly known that articular chondrocytes require a 3D environment to preserve their polygonal morphology and to deposit their synthesized ECM components. Advanced biomaterials composed of cartilage-specific ECM components to induce particular cell responses (Elisseeff et al. 2006) together with extended exogenous Sox9 exposure may improve the chondrocytes’ stability and prevent hypertrophy. The application of biofunctionalized materials may also provide a solution to the long culture period of iPSC-based chondrocyte differentiation.

Although native articular chondrocytes express a basal type I collagen concentration, transiently induced fibroblasts continued to synthesize high levels of type I collagen. Consequently, the cartilage-like tissue constructs resemble rather fibrocartilaginous than AC tissue. The occurrence of type I collagen can be explained by (1) the presence of non- or partly converted fibroblasts or (2) the reconversion of the cells back to the fibroblast lineage. In both cases, it is assumed that epigenetic remodelling was not sufficient to erase and block fibroblast-specific gene expression. A potential solution is the inclusion of an epigenetic activation phase that may lead to epigenetic erasure of fibroblastic differentiation marks before the cells are exposed to chondrogenic development clues. This strategy minimizes epigenetic memory, and it makes the cells more susceptible to reprogramming factors. More importantly, it allows the cells to expand during the intermediate phase, which is not possible in lineage-instructive reprogramming.

A last generic discussion point regarding both approaches is the lack of proper controls and epigenetic screening in the reviewed studies. Obviously, both iPSC-derived and fibroblast-derived chondrogenic cells have to be safe and functional, meaning that genetic aberrations must be minimized and functional epigenetic characteristics of the starting cell type (i.e. fibroblasts) have to be absent. Currently, these features are barely studied before and after initiation of in vitro chondrogenic differentiation. Additionally, the derived chondrogenic cells are compared to native articular chondrocytes only in a few studies. Future research should include examination, by for example genome and epigenome sequencing, to assess the quality of the derived chondrocytes for clinical application.

## Conclusion

Nowadays, reprogramming strategies are of high interest in clinical research to develop a new cell source for regenerative medicine and tissue engineering. Both approaches elaborated in this article allow the use of abundant, accessible and autologous somatic cells for cartilage formation, yet are still inefficient and time- and cost-consuming. Direct lineage reprogramming provides a slight advantage over these aspects compared to the iPSC detour. However, the risk of fibrocartilaginous tissue due to residual fibroblastic cells is a serious consideration for bringing this approach to the clinic.

In our opinion, the iPSC detour for cartilage regeneration is currently closer in the vicinity of the clinic due to more progress and attention in the safety- and efficacy-related aspects of the method and derived cells. For instance, the risk of teratoma formation has been minimized by improvement of the approach in the absence of oncogenes, contrary to direct lineage reprogramming that still requires constitutive transgene expression. Also, expansion of the cell number, needed for in vivo cartilage regeneration, is easier in the presence of a pluripotent cell state during the reprogramming process. Lastly, directed differentiation of iPSCs allows the application of cell isolation techniques to derive a homogenous cell population that in turn contributes to the safety and functionality of the cells. Overall, more academic attention is needed for direct lineage reprogramming in order to compete with the iPSC detour for clinical purposes in the field of cartilage repair.

Finally, many scientists acknowledge the importance of epigenetic memory in chondrocyte safety and functionality. For this reason, it remains significant to include epigenetic profiling of reprogrammed cells and compare this to appropriate control groups. The future challenge is to tackle current drawbacks by further research into the optimal reprogramming and culture conditions while holding the clinical objectives in mind. This includes studies focussed on the identification and determination of small molecules, chemical components and (hypoxic) 3D microenvironments that promote chondrogenic reprogramming and prevent tumourigenic, fibroblastic and hypertrophic activities.

## Abbreviations

AA2P: Ascorbic acid 2-phosphate
ACAN: Aggrecan
ACI: Autologous chondrocyte implantation
BMP: Bone morphogenetic protein
c-Myc: Myelocytomatosis oncogene
COL2A1: Collagen type II alpha 1
DF: Dermal fibroblasts
EB: Embryoid body
ECM: Extracellular matrix
EMT: Epithelial-to-mesenchymal transition
ESC: Embryonic stem cell
FBS: Foetal bovine serum
FCM: Flow cytometry
FGF-2: Fibroblast growth factor 2
GAG: Glycosaminoglycan
GDF5: Growth and differentiation factor 5
GSK3: Glycogen synthase kinase 3
HDAC: Histone deacetylase
iPSC: Induced pluripotent stem cell
Klf4: Krüppel-like factor 4
MET: Mesenchymal-to-epithelial transition
MFC: Microfracture
MMP13: Matrix metallopeptidase 13
MSC: Multipotent mesenchymal stromal cell
Nanog: Nanog homeobox
Oct4: POU class 5 homeobox 1
PDGF: Platelet-derived growth factor
PG: Proteoglycan
PSC: Pluripotent stem cell
PTHrP: Parathyroid hormone-related protein
RUNX2: Runt related transcription factor 2
SB431542: TGF-β inhibitor
Sox2: Sex determining region Y box 2
TF: Transcription factor
TGF- β: Transforming growth factor-β
VitC: Vitamin C
VPA: Valproic acid
WNT3A: Wingless-type family member 3a

## Footnotes

1 Reprogrammed cells are selected with a neomycin resistance gene and β-galactosidase reporter incorporated into F-box protein 15 (Fbx15), a target gene for Oct3/4 that is dispensable for pluripotency.
